# Erie County Medical Society—Annual Meeting and Election of Officers

**Published:** 1868-01

**Authors:** T. M. Johnson

**Affiliations:** Secretary


### Erie County Medical Society—Annual Meeting and Election of Officers.

At the Annual Meeting of the Erie County Medical Society, held at their rooms on the 14th instant, Dr. Frank W. Abbott read a paper on "The Sources of Muscular Power," for which a vote of thanks were tendered him, and a copy requested for publication.

Dr. J. R. Lothrop, the retiring President of the Society, delivered an able and interesting valedictory address. A vote of thanks was also tendered Dr. L., and a copy of the address requested for publication.

A committee appointed for the purpose, consisting of Drs. William Gould, E. R. Barnes and M. G. Potter, submitted the following preamble and resolutions:

WHEREAS, Dr. M. E. Shaw has been removed by death, while exposed to unusual dangers in the discharging of his duties as a physician, and under circumstances peculiarly calculated to enlist our sympathy; therefore

*Resolved*, That while we bow to the will of Providence, we deeply regret the loss of one who, in his brief career, showed himself faithful to the discharge of his duties, and displayed abilities which gave promise of a life of honor and usefulness.

*Resolved*, That we deeply sympathize with the bereaved parents of the deceased, who, by this blow, have been deprived of a son whose qualities of heart and mind were such as to render him peculiarly dear to them.

*Resolved*, That a copy of these resolutions be presented to the family of the deceased.

The resolutions were adopted, after which the following officers were elected for the ensuing year:

*President*—Dr. John Boardman.
*Vice-President*—Dr. O. K. Parker.
*Secretary*—Dr. M. G. Potter.
*Treasurer*—Dr. William Ring.
*Librarian*—Dr. James B. Samo.
*Primary Board*—Drs. Thomas M. Johnson, James B. Samo, J. S. Smith.
*Board of Censors*—Drs. S. W. Wetmore, S. F. Mixer, Thomas Lothrop, P. H. Strong, John Hauenstein.

Drs. E. R. Barnes, of Buffalo, and A. R. White, of Tonawanda, were elected to membership upon compliance with the by-laws.

T. M. JOHNSON, M. D., *Secretary.*

